# The Effects of Working Memory Updating Training in Parkinson’s Disease: A Feasibility and Single-Subject Study on Cognition, Movement and Functional Brain Response

**DOI:** 10.3389/fpsyg.2020.587925

**Published:** 2021-01-13

**Authors:** Lois Walton, Magdalena Eriksson Domellöf, Carl-Johan Boraxbekk, Erik Domellöf, Louise Rönnqvist, David Bäckström, Lars Forsgren, Anna Stigsdotter Neely

**Affiliations:** ^1^Department of Social and Psychological Studies, Karlstad University, Karlstad, Sweden; ^2^Department of Psychology, Umeå University, Umeå, Sweden; ^3^Umeå Center for Functional Brain Imaging (UFBI), Umeå University, Umeå, Sweden; ^4^Department of Radiation Sciences, Umeå University, Umeå, Sweden; ^5^Danish Research Centre for Magnetic Resonance, Centre for Functional and Diagnostic Imaging and Research, Copenhagen University Hospital, Hvidovre, Denmark; ^6^Institute of Sports Medicine Copenhagen (ISMC), Bispebjerg Hospital, Copenhagen University, Copenhagen, Denmark; ^7^Department of Pharmacology and Clinical Neuroscience, Umeå University, Umeå, Sweden; ^8^Engineering Psychology, Luleå University of Technology, Luleå, Sweden

**Keywords:** Parkinson’s disease, cognitive training, cognition, working memory, movement kinematics, functional magnetic resonance imaging

## Abstract

In Parkinson’s disease (PD), the fronto-striatal network is involved in motor and cognitive symptoms. Working memory (WM) updating training engages this network in healthy populations, as observed by improved cognitive performance and increased striatal BOLD signal. This two-part study aimed to assess the feasibility of WM updating training in PD and measure change in cognition, movement and functional brain response in one individual with PD after WM updating training. A feasibility and single-subject (FL) study were performed in which patients with PD completed computerized WM updating training. The outcome measures were the pre-post changes in criterion and transfer cognitive tests; cognitive complaints; psychological health; movement kinematics; and task-related BOLD signal. Participants in the feasibility study showed improvements on the criterion tests at post-test. FL displayed the largest improvements on the criterion tests and smaller improvements on transfer tests. Furthermore, FL reported improved cognitive performance in everyday life. A shorter onset latency and smoother upper-limb goal-directed movements were measured at post-test, as well as increased activation within the striatum and decreased activation throughout the fronto-parietal WM network. This two-part study demonstrated that WM updating training is feasible to complete for PD patients and that change occurred in FL at post-test in the domains of cognition, movement and functional brain response.

## Introduction

Parkinson’s disease (PD) has traditionally been defined as a predominantly motor disorder due to dopamine (DA) depletion, which has led to cognitive symptoms being under recognized in the clinical practice (Barone et al., 2011). Cognitive deficits are however a common and disabling symptom of PD with 62% of newly diagnosed PD patients showing deficits on at least one neuropsychological test and 30% meeting criteria for mild cognitive impairment (MCI) (Williams-Gray et al., 2007; Elgh et al., 2009). PD patients with cognitive symptoms report lower overall quality of life (Leroi et al., 2012), display a faster decline in cognitive abilities (Broeders et al., 2013) and are more likely to be diagnosed with PD dementia at a 5-year follow-up (Domellöf et al., 2015).

Some motor and cognitive symptoms, such as bradykinesia and working memory (WM), show a positive correlation (Domellöf et al., 2011) and share an underlying reliance on DA availability in the fronto-striatal network (Kehagia et al., 2012). Decreased activity and connectivity in this network has been linked to several cognitive and motor deficits (Leisman and Melillo, 2013; Xu et al., 2016). Standard treatment in the form of dopamine replacement therapy aims to increase dopaminergic availability, yet does not lead to clear benefits in cognitive functioning (MacDonald and Monchi, 2011; Roy et al., 2018). Non-pharmacological interventions that aim to enhance DA are therefore interesting candidates in the PD population.

Working memory updating training focuses on monitoring and coding information while replacing old, no longer relevant information with newer, more relevant information (Miyake et al., 2000; Soveri et al., 2017). Brain-imaging studies report that deficits in WM updating are related to lower DA availability and decreased connectivity within the fronto-striatal pathways in both healthy and PD populations (Ekman et al., 2012; Salami et al., 2018). Our group has previously shown that WM updating training leads to improvements in WM updating and near-transfer cognitive tests, as well as an increased striatal BOLD signal in healthy young and older adults (Dahlin et al., 2008a,b). Other studies have reported similar changes in BOLD signal in striatal and frontal areas in healthy populations after WM updating training (Kühn et al., 2013; Buschkuehl et al., 2014). Furthermore, the striatal BOLD signal has shown to be positively correlated with dopamine release (Schott et al., 2008) and WM updating training studies have revealed that enhanced DA release occurs after WM updating training in a group of young healthy adults (Bäckman et al., 2011, 2017). Recently, Fellman et al. (2018) conducted a WM updating training study in a PD population and reported improvements in trained and near-transfer cognitive tasks. The current investigation intended to assess if WM updating training is feasible for PD patients to complete and extend the findings of Fellman et al. (2018) by assessing change in cognition, motor, psychological health and BOLD response in one individual with PD after WM updating training. The following research questions were addressed: (1) Is WM updating training feasible for PD patients to complete?; (2) Can we measure change in cognitive performance, goal-directed upper-limb movement, psychological health and functional brain response in one individual with PD after completion of WM updating training.

## Feasibility Study

### Materials and Methods

#### Participants

Patients were recruited from a prospective population-based study on idiopathic parkinsonism, i.e., the NYPUM project at Umeå University Hospital, Sweden. Inclusion criteria were definite or probable PD according to UKPDSBB-criteria (Gibb and Lees, 1988), Mini Mental State Examination above 24 and no additional severe diseases or psychological disorders. Thirteen participants were invited, four agreed to participate. One participant did not complete the intervention due to personal reasons. One of the remaining three participants was diagnosed with a MCI according to the criteria of the Movement Disorder Society (Litvan et al., 2012). All participants received standard treatment. See Table 1 for the participants’ demographic and clinical characteristics.

**TABLE 1:** Demographic and clinical characteristics of the participants.

**Measures**	**PD 1**	**PD 2**	**PD 3**
Age	79	70	59
Gender/sex	Male	Male	Male
Disease duration (months)	10	12	39
Hoehn and Yahr stage	2.0	2.0	2.5
UPDRS III	33	43	33
MMSE	29	29	29
MCI	No	Yes	No
LEDD	600	504	335

*UPDRS III, Unified Parkinson Disease Rating Scale section III (motor part); MMSE, Mini Mental State Examination; MCI, mild cognitive impairment; LEDD, total levodopa equivalent daily dose calculated according to Tomlinson et al. (2010).*

#### Design

The study has a non-experimental pre- and post-test design with multiple assessments of the target behavior across the intervention in three individuals with PD (Tate et al., 2016).

#### Intervention

**FIGURE 1:**
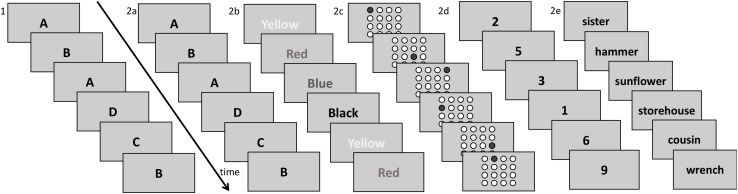
Overview of the WM updating training which consisted of the criterion training test (see part 1) and five running span training tasks with different types of stimuli (part 2a-2e).

The WM updating training was a computerized program consisting of six different updating tasks (Dahlin et al., 2008a,b), see Figure 1. The participants performed the training at Umeå University three times a week (5 weeks in total) on individual computers while seated in the same room. Each session lasted 45 min. Further details on the intervention can be found in the Supplementary Material.

#### Outcome Measures

Following the guidelines by Bowen et al. (2010), feasibility outcome measures assessed the acceptability and practicality of the WM updating training through a short questionnaire. This consisted of open-ended questions pertaining to acceptability through asking about the set-up and execution of the training. Practicality was assessed via questions related to the difficulty level and completion time of the training. Attendance was collected at every training session in order to measure compliance.

Training gain was measured via two versions of the Letter Memory test (referred to as the criterion test and the criterion training test) to allow comparison with our previous work (Dahlin et al., 2008b; Sandberg et al., 2014). The criterion test was administered at pre- and post-test. The criterion training test was used to measure gain across sessions (number of correct recalled 4-letter sequences). The participants’ learning curves were compared with a group of 11 younger (mean age = 24) and 12 older (mean age = 68) healthy participants that completed the same WM updating training (Dahlin et al., 2008a). Self-perceived improvement on the training tasks and everyday functioning was assessed post-training through a questionnaire with a 5-point Likert scale. Further details on the outcome measures can be found in the Supplementary Material.

### Results

The training was considered feasible since three of the four participants completed the full training program. Participants reported acceptability toward the WM updating training through expressing satisfaction with the set-up and execution of the training. Concerning the practicality of the intervention, the participants stated the training was of a satisfactory difficulty level and completion time. More specifically, participants perceived the training as challenging and stimulating, as well as that the time to complete each session was of an adequate length. One participant also indicated that 15 on-site sessions over 5 weeks was rather intensive.

**FIGURE 2:**
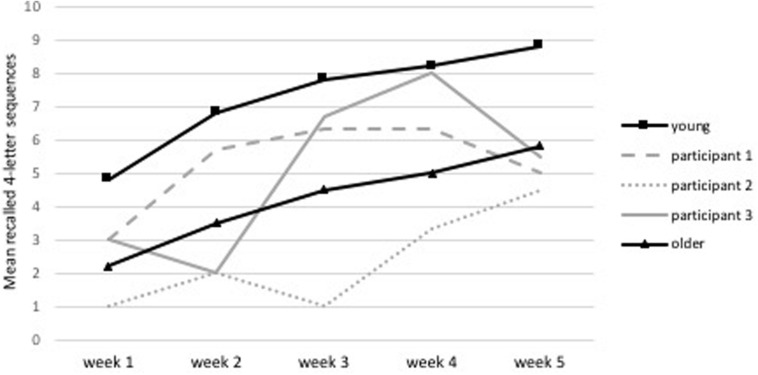
Learning curve during training for the mean recall of 4-letter sequences during the criterion training test. The three PD patients are compared to a healthy young and older population (Dahlin et al., 2008a).

**FIGURE 3:**
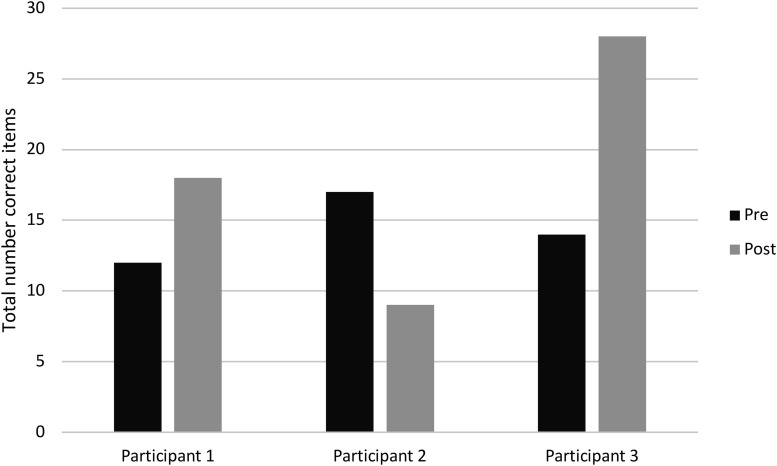
Training gain seen as improved performance in the criterion test (feasibility study).

All participants displayed improved performance on the criterion training test, as seen in Figure 2 where their results are compared to healthy young and older participants who completed the same WM updating training in a previous study (Dahlin et al., 2008a). The learning curve of the patient with an MCI (i.e., participant 2 in Figure 2) is situated under the healthy older population’s learning curve, yet also displays an improvement. Moreover, two participants displayed improvements on the criterion test at post-test, see Figure 3. All participants stated self-perceived improvement on the trained task and participants 1 and 3 reported improvements in everyday life.

### Summary

This feasibility study suggested that the WM updating training is feasible for PD patients to complete. Furthermore, two participants demonstrated improvements in the criterion test and experienced subjective cognitive improvements in everyday life at post-test. Moreover, all participants displayed positive learning curves across training, resembling those of a healthy older population that had completed the same training previously (Dahlin et al., 2008a). Interestingly, the participant with an MCI displayed no improvement on the criterion test, yet did have a positive learning curve over time. As the criterion test had a time constraint, it is likely that it was too difficult and was therefore unable to capture the improvements seen on the criterion training test. However, the learning curve suggested that even participants with an MCI may benefit from WM updating training.

Considering that nine of the 13 invited participants declined participation in the study due to a lack of motivation and/or time constraints, the training was adapted to a home-based version. This allows for a more flexible training program whereby the participant does not need to travel to the study setting three times per week to perform the training, as was the case in this feasibility study.

In conclusion, these promising results motivated the single-subject study in which additional outcome measures beyond cognitive functioning were added to capture changes in other domains often related to the fronto-striatal network, i.e., motor functioning and striatal BOLD response. Thereby, the single-subject study is addressing the broader effects of WM updating training in one individual with PD.

## Single-Subject Study

### Materials and Methods

#### Participant

The participant, referred to as FL, is female, right-handed, 47 years old, employed, with an academic education, diagnosed with PD according to the UKPDSBB-criteria (Gibb and Lees, 1988), less than 2 years since diagnosis, HoY stage I, no other diseases in the CNS and intact cognitive function based on verbal tests (WAIS-IV).

#### Design

This exploratory single-subject study has a non-experimental pre- and post-test design with multiple assessments of the target behavior across the intervention (Tate et al., 2016).

#### Intervention

A web-based training program was conducted unsupervised at home 4–5 times a week for a total of 30 training sessions. The training program was similar to the feasibility study’s training program. Further details can be found in the Supplementary Material.

#### Outcome Measures and Data Analysis

##### Cognition

The cognitive test battery consisted of nine tests with different levels of overlap with the training tasks. These tests were divided into four categories, based on a meta-analysis on the effects of WM training (Melby-Lervåg et al., 2016): (1) the criterion tests, i.e., tests that are close to identical to the training tasks; (2) near transfer tests, i.e., tests that are not part of the training, yet aim to measure WM updating; (3) intermediate transfer tests, i.e., tests that measure other aspects of WM: maintenance and manipulation of information, inhibition and shifting; and (4) far transfer tests, i.e., tests measuring untrained cognitive abilities such as episodic memory, mental speed and reasoning.

To measure training gain, the same criterion tests as in the feasibility study were used. The criterion test was performed at pre- and post-test. The criterion training test was part of each training session to examine gain across training. Two near transfer tests were used to assess WM updating, i.e., the Number Memory test and n-back. Four intermediate transfer tests were performed, i.e., Digit Span and Letter-Number sequencing, both from WAIS-IV (Wechsler, 2008) to assess the ability to maintain and manipulate information in WM, while inhibition and shifting were measured with the Color-Word interference test and the Trail Making test, both from D-KEFS (Delis et al., 2001). Far transfer tests consisted of Buschke’s Selective Reminding Test (Buschke, 1973), Matrix Reasoning (WAIS-IV), Coding (WAIS-IV) and TMT-2 (D-KEFS), to measure episodic memory, problem solving and speed of processing, respectively. All details concerning these tests can be found in the Supplementary Material.

To analyze the cognitive data, a baseline population consisting of 24 individuals with PD aged 45–65 (mean age = 59) with a disease duration shorter than 5 years was used as a norm group to compare FL’s results with. *Z*-scores for all measurements were calculated through using the mean and standard deviation from this population. *Z*-score change was calculated with the following equation:

z-score⁢change=post⁢-⁢test⁢z-score-pre⁢-⁢test⁢z-score

As FL is an early onset PD patient, the norm group does not match in terms of age. Therefore, the magnitude of the *z*-scores and the *z*-score change will be discussed from a visual perspective in which we are especially interested in the relative difference between the *z*-score change in the criterion tests compared to near, intermediate and far transfer tests. *Z*-scores for timed tasks were reversed, so that a positive *z*-score indicates a faster speed than the norm group. The baseline population did not perform the Letter Number Sequencing test, therefore WAIS-IV norms were used instead.

##### Subjective cognitive complaints and psychological health

Subjective cognitive complaints were assessed with the Prospective Retrospective Memory Questionnaire (PRMQ). The Hospital Anxiety Depression (HAD) scale was used to assess symptoms of depression and anxiety, the Parkinson’s Disease Questionnaire (PDQ-39) to assess function and wellbeing related to PD, and the Checklist Individual Strength (CIS) was used to examine fatigue. Further details on these questionnaires can be found in the Supplementary Material. Results on these measurements were analyzed through using the mean and standard deviation from the same norm group as previously stated to calculate *z*-scores. *Z*-scores for the CIS and PRMQ were reversed, so that a positive *z*-score change indicates an improvement in the measured domain.

##### fMRI image acquisition and analysis

A 3T Discover MR750 General Electric (GE) scanner equipped with a 32-channel head coil was used for data collection with the following parameters: echo time = 30 ms; repetition time = 2,000 ms; flip angle = 80°; field of view = 25 × 25 cm; matrix size = 96 × 96; slice thickness = 3.4 mm; number of slices = 37. In scanner tests consisted of the criterion test and the *n*-back test.

Before image acquisition, 10 dummy scans were collected and discarded. A whole brain analysis was applied to the fMRI data. Pre-processing and analysis was performed with SPM12^1^. All images were corrected for slice timing, realigned and unwrapped to correct for head movements. No normalization was performed. The final voxel size was 2 × 2 × 2 mm.

A general linear model was applied for each paradigm (Letter Memory and *n*-back) respectively. Movement parameters were added as nuisance regressors. The contrast (updating vs rest) was used to assess the pre to post changes during the Letter Memory test. Two contrasts (2-back − 1-back) and (3-back − 1-back) were used to assess the pre to post changes during the n-back test. A conservative threshold of *p* < 0.01 FWE corrected was applied.

##### Goal-directed movement

Hand tremor and goal-directed upper-limb movement were examined at pre- and post-test. Postural and resting tremor was measured unilaterally (right and left) and bilaterally (both sides) when seated with the arm extended in the air or flexed with elbow support. The measurement time of each trial was pre-set to 10 s, and generated 10 individual arm/hand movements from each side at pre- and post-test (in total 40). Goal-directed upper-limb movement was assessed during a task where the participant pressed three buttons sequentially with the left or right index finger (unimanual) or with both index fingers simultaneously (bimanual) (Johansson et al., 2012). The task was performed in two directions, i.e., vertically or horizontally. Three trials were performed for each condition and direction in a randomized, counterbalanced order. The participant was fitted with a set of spherical passive markers, affixed to the forehead (two markers), shoulders, elbows, wrists, and index fingers. Movement kinematics were recorded by a six-camera optoelectronic registration system (ProReflex, Qualisys Inc., Gothenburg, Sweden). Data were sampled at a frequency of 120 Hz. Onset latency was defined as the difference in frames between start of measurement (synchronized with an auditory signal denoting start for the participant) and movement onset in terms of wrist marker tangential velocity attaining or exceeding 20 mm/s. A movement unit (MU) was defined as an accumulated velocity increase/decrease of at least 20 mm/s with an acceleration/deceleration exceeding 5 mm/s^2^ (von Hofsten, 1991).

All kinematic data was smoothed prior to analysis using a second-order 10 Hz dual-pass Butterworth filter, and analyzed in MATLAB (The Math-works Inc.). Tests of homogeneity of variance and normality between-tests supported that the outcome data were normally distributed at both pre-and post-test. To analyze possible pre- to post-test differences, a 2 (test: pre/post) × 2 (side: right/left) × 2 (task: bi/unimanual) factorial design was used. Each dependent variable in focus was run in separate tests with *p* < 0.05 as the level of significance. The statistical analysis was made by use of STATISTICA software (StatSoft13). In total 104 individual (R + L) arm/hand recordings were included in the analyses (64 goal-directed task recordings and 40 10-s baseline-tremor task recordings).

## Results

### Training Gain in Criterion and Transfer Tests

An improvement on the criterion test was seen by an increase in the total number of items correct and number of correct recalled 4-letter sequences with *z*-score changes of 2.96 and 2.84, respectively. FL displayed a similar learning curve to that of the young and older healthy population who had performed the same WM updating training previously (Dahlin et al., 2008a). The curve starts in between the performance levels of the young and old though quickly approaches the performance levels of the young to then slightly drop off. Some fluctuations can be seen closer to the end of the training, which can be observed in Figure 5.

Substantial near transfer effects were visible through an increase in the total number of items correct and number of correct recalled 4-digit sequences in the Number Memory test with corresponding *z*-score changes of 1.39 and 1.3. Total score over all three conditions of the n-back tests did not show any changes due to a ceiling effect, yet the 3-back condition did demonstrate a small improvement with a *z*-score change of 0.26.

**FIGURE 4:**
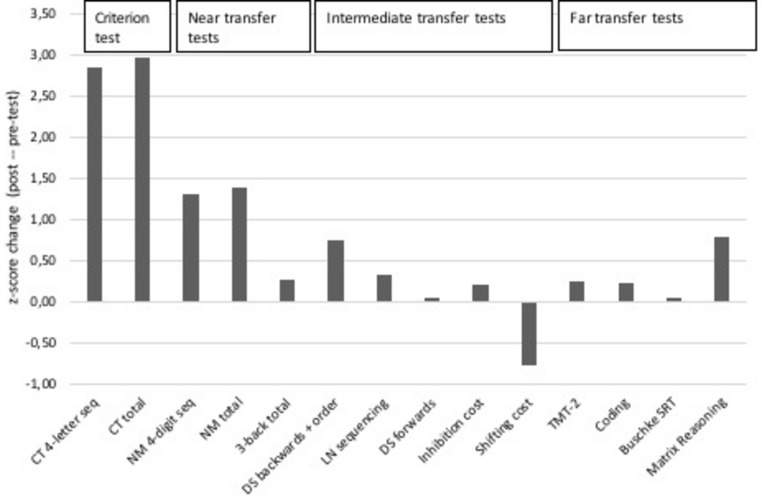
Training gain seen as improved performance on the criterion test, near, intermediate and far transfer tests. Note. CT 4-letter seq: number of 4-letter sequences recalled correctly in criterion test; CT total: total items correct in the criterion test; NM 4-digit seq: number of 4-digit sequences recalled correctly in the Number Memory test; NM total: total items correct in the Number Memory test; 3-back total: total score on the 3-back test (i.e., hits minus false alarms); DS backwards + order: total number of correct items from the two subtests of Digit Span (WAIS-IV); LN sequencing: total items correct on the Letter Number Sequencing test (WAIS-IV); DS forwards: total number of correct items on the forwards span subtest of Digit Span (WAIS-IV); Inhibition cost: time taken (seconds) to complete incongruent trials as compared to reading colored words condition (D-KEFS); Shifting cost: the difference in time (seconds) taken to complete TMT part 4 as compared to TMT part 2 (D-KEFS); TMT-2: total time to complete the Trail Making Test subtest 2 (D-KEFS); Coding: total items correct (WAIS-IV); Buschke SRT: total recall score from Buschke’s Selective Reminding Test; Matrix Reasoning: total items correct.

Small to moderate intermediate transfer effects were seen in tests that measure active WM with *z*-score changes of 0.74 (Digit Span backwards + sequencing) and 0.33 (Letter Number Sequencing). A test that measures passive WM did not show any change, with a *z*-score change of 0.00 (Digit Span forwards). The inhibition cost indicated a negligible improvement with a *z*-score change of 0.19, while the shifting cost displayed a decline with a *z*-score change of −0.78. An overview of these *z*-scores can be observed in Figure 4.

**FIGURE 5:**
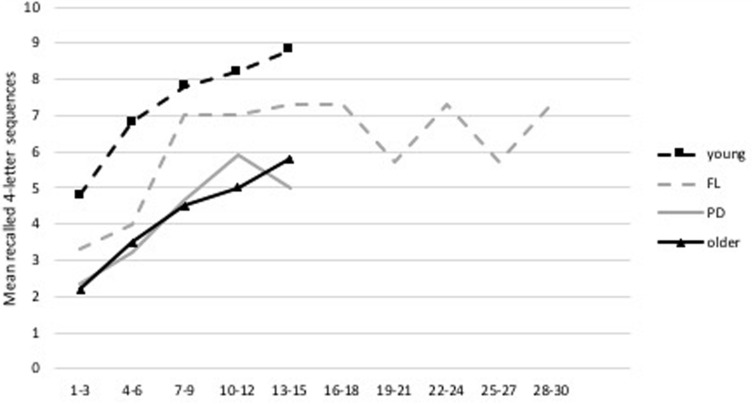
Learning curve during WM updating training. Average taken over three sessions (e.g., training session 1, 2, and 3) of the number of correct recalled 4-letter sequences during the criterion training test. FL’s curve is depicted together with a young and older healthy population performing the same WM updating training as derived from Dahlin et al. (2008a) and the average learning curve of the three PD patients from the feasibility study.

A moderate improvement was observed in a test measuring problem solving (Matrix Reasoning) with a *z*-score change of 0.78. Small gains were seen in tests measuring speed of processing with *z*-score changes of 0.24 (TMT-2) and 0.21 (Coding). Episodic memory performance did not show any difference with a *z*-score change of 0.00 (Buschke’s Selective Reminding Test). Figure 4 provides and overview of these results.

### Subjective Cognitive Complaints and Psychological Health

**FIGURE 6:**
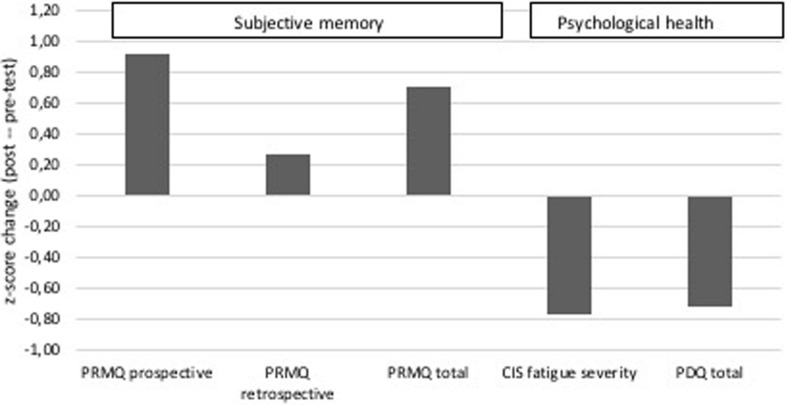
FL’s z-scores on questionnaires on cognitive complaints and psychological health at pre- and post-test. Depicted are the prospective subscale, retrospective subscale and total score of the PRMQ self-rating scale; the fatigue severity subscale of the CIS questionnaire; and the total score on the quality of life questionnaire specific for PD (PDQ).

FL reported a moderate improvement in overall subjective cognitive abilities at post-test with a *z*-score change of 0.70. A moderate improvement was seen in the subdomain of prospective memory, with a *z*-score change of 0.92. FL did not report depression or anxiety levels that were over the cut-off for advised further follow-up at pre- or post-test. However, FL reported more fatigue at post-test with especially higher levels of fatigue severity symptoms with a *z*-score change of −0.77. Moreover, FL reported a decrease in quality of life at post-test, with a *z*-score change of 0.73. Figure 6 provides an overview of these results.

### Goal-Directed Movement and Baseline Hand-Tremor

**FIGURE 7:**
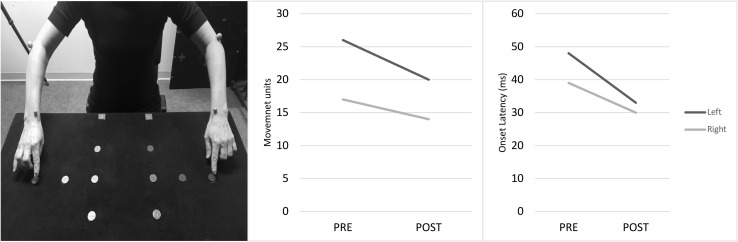
Set-up of the goal-directed movement task (left), FL’s movement units (middle) and onset latency (right) at pre- and post-test.

A main effect of test session was observed for the goal-directed movement onset latencies (from wrist markers), displaying a shorter and more consistent onset latency at post-test (*M* = 258 ms, *SD* = 88 ms) in comparison to pre-test (*M* = 362 ms, *SD* = 172 ms), *F*(1, 56) = 9.48, *p* = 0.003. Thereby, indicating a shorter movement initiation and better movement pre-planning after WM updating training, depicted in Figure 7. Additionally, a main effect of test session was found regarding number of MUs for both movements of the wrist (*M*^*pre*^ = 14.6, *SD* = 7.6; *M*^*post*^ = 11.3, *SD* = 5.9), *F*(1, 56) = 4.83, *p* = 0.03, and the index-finger (*M*^*pre*^ = 21.8, *SD* = 12.1; *M*^*post*^ = 16.6, *SD* = 6.8), *F*(1, 56) = 5.72, *p* = 0.02, showing smoother (less segmented) and more organized goal-directed hand–finger movements after completion of the WM updating training, as seen in Figure 7. Furthermore, no significant main effect of test session was displayed regarding the baseline hand-tremor outcomes for number of movement segmentation (MUs/10s trials), *F*(1, 36) = 2.56, *p* = 0.118 (*M*^*pre*^ = 124, *SD* = 17; *M*^*post*^ = 131, *SD* = 27), nor any significant effect of side, *F*(1, 36) = 3.04, *p* = 0.089 (*M*^*Right*^ = 132, *SD* = 20; *M*^*Left*^ = 125, *SD* = 26). Further details on the kinematic results can be found in the Supplementary Material.

### Brain Imaging

**FIGURE 8:**
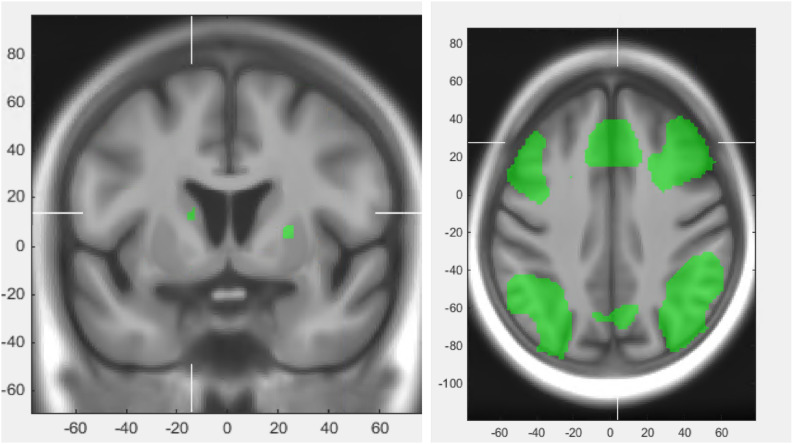
Increase in BOLD activation in striatum during performance of the Letter Memory test at post-test (left) and the general fronto-parietal network during performance of Letter Memory test and n-back (right).

Following training, decreased activation throughout the fronto-parietal WM network was observed during the Letter Memory test. This includes decreased activation within the dorso-lateral prefrontal cortex (DLPFC), the anterior cingulate cortex (ACC), the inferior frontal gyrus (IFG) and the posterior parietal cortex (PPC). During n-back, a similar pattern was observed with decreased activity within parts of the fronto-parietal network including DLPFC, ACC, IFG and PCC, as depicted in Figure 8. However, the 2-back contrast and the 3-back contrast displayed slight differences. The 3-back contrast displayed alongside with the decreased activation, increased activation in parts of the DLPFC, whilst the 2-back contrast did not show such increases.

For both the letter memory and the n-back test, increased activation within the striatum was observed after training, mainly in the putamen.

## Discussion

The objective of this two-part study was to evaluate the feasibility of WM updating training in three individuals with PD and measure behavioral and cerebral change after completion of WM updating training in one participant with PD. We assessed change in cognition, psychological health, functional brain response and upper-limb goal-directed movement.

Our main results showed that the WM updating training was feasible to complete and was accompanied with large improvements on the criterion test. Furthermore, small to moderate improvements on near and intermediate transfer tests, decreased activation in the fronto-parietal WM network and increased activation in the striatum were seen in FL after training. Moreover, smoother, better organized upper-limb movements and improved subjective cognitive performance were assessed at post-test in FL.

To increase our knowledge of how to best support cognitive functioning in PD is of great clinical relevance and a key priority in the field. Process-based cognitive interventions, such as WM and Executive Functioning training, have attracted much scientific interest during the last two decades as a mean to improve cognitive health in normal and pathological aging (Gavelin et al., 2020). Although, the empirical findings are promising it is still hotly debated whether these training paradigms result in far-transfer effects and impact everyday life (Au et al., 2015; Melby-Lervåg et al., 2016; Soveri et al., 2017; Nguyen et al., 2019; Gavelin et al., 2020).

Within the PD population, most previous studies on the effect of cognitive training have employed multi-domain training programs (Walton et al., 2017). Even though these studies have shown improvements in cognition, changes in functional brain response and enhanced quality of life (Leung et al., 2015), the wide scope of such interventions leads to difficulties in drawing causal relationships between a specific part of the training and the outcome. In order to develop patient-focused, yet cost-efficient cognitive rehabilitation, studies must examine the etiology of cognitive disorders in PD and attempt to influence these mechanisms through specific cognitive training. Unique to the current study is its groundings in the fronto-striatal hypothesis of PD, whereby a decrease in activity and connectivity in the dopaminergic fronto-striatal network has been linked to both several cognitive and motor deficits in PD (Kehagia et al., 2012; Leisman and Melillo, 2013; Xu et al., 2016). WM updating training has shown to activate this network in healthy populations, as seen by decreases in BOLD response in frontal and parietal areas, as well as changes in the striatal BOLD signal after training (Hempel et al., 2004; Dahlin et al., 2008b; Schneiders et al., 2012; Kühn et al., 2013). Our results indicated a similar pattern of change in functional brain response at post-training in FL with increased activation in the striatum and decreased activation in the fronto-parietal network, tentatively suggesting that WM updating may have engaged the fronto-striatal pathway in FL. Prior studies have shown that WM capacity is related to dopamine synthesis in the striatum (Cools et al., 2008) and increased dopamine release in the striatum has been measured after WM updating training (Bäckman et al., 2011, 2017). Considering that striatal BOLD response has been shown to be correlated with the release of dopamine (Schott et al., 2008), one account of the present fMRI findings is that the increased BOLD response in the striatum may be linked to release of dopamine.

Results from the kinematic assessment showed an improvement of the goal-directed, sequential hand/finger movement executions at post-test in FL. Movements were less segmented, better organized and with shorter onset latency times post-training. Previous studies have reported shorter movement onset times after levodopa administration (Haslinger et al., 2001; Spay et al., 2019) and goal-directed movement is reliant on the fronto-striatal network (Redgrave et al., 2010). In contrast, tremor is primarily associated with activity in the cerebello-thalamo-cortical loop (Dirkx et al., 2016). The combination of the improvements in goal-directed movement together with the lack of change in FL’s tremor might therefore be another indication that the WM updating training was specifically targeting the fronto-striatal network in FL.

All participants showed large improvements on the criterion test and positive learning curves during training. Interestingly, these learning curves portrayed the same progression, regardless of age or the fact that one participant had an MCI and another was an early onset PD patient. Hence the training effect seems to have been similar in this diverse group of PD patients. This is partly in line with other studies that showed cognitive training to be beneficial for PD patients with MCI (Reuter et al., 2012; Petrelli et al., 2014). Moreover, FL displayed a substantial improvement in a near transfer test, which is in line with previous studies on WM updating training in healthy populations (Dahlin et al., 2008b; Waris et al., 2015; Soveri et al., 2017; Linares et al., 2018). Intermediate transfer tests showed small to moderate gains in active WM, whilst tests measuring passive WM, inhibition and shifting failed to show improvement in FL. Such selective transfer effects to active WM tasks in comparison to passive WM tasks is in keeping with prior research (Waris et al., 2015; Fellman et al., 2018), as have improvements in active WM been associated with increased dopaminergic availability (Grogan et al., 2018). Far transfer effects were absent in FL, except for a moderate improvement on an indicator of fluid intelligence. This finding is in line with studies displaying small effects of WM updating training on fluid intelligence (Au et al., 2015; Soveri et al., 2017). Overall, these cognitive results are corroborated by other WM updating training studies in which larger effects are seen in tests similar to the training tasks, while far transfer effects remain rare (Waris et al., 2015; Soveri et al., 2017; Fellman et al., 2018).

Besides the changes in objective cognitive test performance, FL expressed less cognitive failures in daily life, especially within the domain of prospective memory. Since memory for future intentions are of great importance to everyday life, this finding may have functional relevance. Studies have shown that PD patients perform better on prospective memory tasks when the need to update and monitor information is reduced (Kliegel et al., 2011). The improvement that FL reported in prospective memory might thereby be a consequence of increased WM updating performance. Furthermore, FL reported more fatigue and lower quality of life at post-test, most likely due to disease progression. It is noteworthy that even when experiencing such difficulties, FL still reported improved cognitive performance in everyday life at post-test. This lack of change in quality of life has also been found in studies assessing other types of cognitive training in PD (Leung et al., 2015).

The following limitations of the study can be noted. Firstly, as is always the case with non-experimental single-subject studies, statements concerning causality cannot be made. Furthermore, the norm group was older than FL, who is an early onset patient, most likely leading to *z*-score overestimation. Therefore, caution is warranted when interpreting FLs level of performance. Moreover, the single-subject study lacks multiple measurements before the start of the WM updating training to ensure a more reliable baseline measurement. Also, motivation and expectation of participants have been shown to influence cognitive training outcomes (von Bastian and Oberauer, 2014), which were not controlled for in this study.

In conclusion, this feasibility and single-subject study aimed to assess the changes seen in participants with PD in regard to cognition, motor and BOLD response after completing a WM updating training program. The combination of these multi-modal findings presents a pattern that might imply that the WM updating training engages the fronto-striatal network in FL. Caution is warranted though, as generalizations to other individuals with PD cannot be made from this single-subject study. Nevertheless, these promising results are a hopeful start to further investigate the effects of WM updating training in PD through independent replication and randomized controlled trials.

## Data Availability Statement

The raw data supporting the conclusions of this article will be made available by the authors, without undue reservation.

## Ethics Statement

The studies were conducted in accordance with the Declaration of Helsinki and were approved by the Umeå Ethical Review Board (currently the Swedish Ethical Review Authority) (Dnr. 09-049M and Dnr. 2016/110-31). Prior to the start of the studies, all participants provided written informed consent and were informed that they may stop participation at any time during the study.

## Author Contributions

LW has made substantial contributions to the conception of the study and wrote the first draft. MD and AS have made substantial contributions to the conception and design of the study as well as the design of the cognitive training program used in these studies. C-JB has had main responsibility for the fMRI analyses. ED and LR for the kinematics analyses. DB and LF for patient recruitment and diagnosis. All authors revised the manuscript as well as read and approved the final manuscript. All authors have agreed both to be personally accountable for the author’s own contributions and to ensure that questions related to accuracy or integrity of any part of the work, even ones in which that author was not personally involved, are appropriately investigated and resolved.

## Conflict of Interest

The authors declare that the research was conducted in the absence of any commercial or financial relationships that could be construed as a potential conflict of interest.

## Footnotes

1 http://www.fil.ion.ucl.ac.uk/spm/software/spm12/
