# Supplementary material for: The Effects of Working Memory Updating Training in Parkinson’s Disease: A Feasibility and Single-Subject Study on Cognition, Movement and Functional Brain Response
Source: Front Psychol. 2021 Jan 13;11:587925. doi: 10.3389/fpsyg.2020.587925 (PMC7838443; doi:10.3389/fpsyg.2020.587925)
Supplement: Supplementary file 1 [file Table_1.docx]

**Supplemental table**

*Description of cognitive outcome measures and questionnaires*

|  | Measure | Neuropsychological domain | Description | Outcome measure |
| --- | --- | --- | --- | --- |
| Criterion test | Letter Memory test | Working Memory updating | The participant was presented with ten counterbalanced lists of the letters A-D with varied lengths (5-15 letters). The letters were presented one at a time, two seconds per letter. Instructions were to recall the four last presented letters in the correct order when the list presentation ended. The participant responded on a computer keyboard using four adjacent keys with the corresponding letters taped over the keys, i.e. A=index finger, B=middle finger, C=ring finger, D=little finger.  Two versions of this test were used in the study, i.e. the criterion training test and the criterion test. The criterion training test was used to measure gain across sessions and was therefore performed at each training instance. The criterion test was administered at pre- and post-test, and had a time constraint of 6 seconds when responding. | Criterion test: Number of correct recalled four-digit sequences (range 0-10) and total correct recalled items (range 0-40)  Criterion training test: number of correct recalled 4-letter sequences (range 0-10) |
| Near transfer tests | Number memory test | Working Memory updating | This test is identical to the criterion test, ﻿yet uses numbers (1−4) instead of letters and consists of 11 lists. | Number of correct recalled four-number sequences (range=0-11) and total correct recalled items (range=0-44) |
|  | n-back | Working Memory updating | This test consists of 27 sequences (nine 1-back, nine 2-back, and nine 3-back) of digits (1−9), which were presented on the screen one digit at a time at a rate of 1500 msec. Each sequence consisted of 10 numbers with four targets and no lures. When a number was presented the participant had to decide if the current number matched the number presented one, two or three positions previously, depending on the instructions before each sequence. The participant used two adjacent keys on the computer keyboard to respond (pressed yes with index finger or no with the middle finger). | Number of hits minus false alarms in each condition (range=0-36) |
| Intermediate transfer tests | Digit Span (WAIS-IV) | Active and passive working memory | The participant was asked to recall digits forwards, backwards and in numerical order. | Number of correctly recalled sequences for Digit Span forwards (range=0-16)  Total score of the number of correctly recalled sequences for Digit Span backwards and numerical ordering (range=0-32). |
|  | Letter-Number sequencing (WAIS-IV) | Active WM | The participant was presented with a series of letters and numbers and asked to first recall the numbers in numerical order followed by the letters in alphabetical order. | Number of correctly recalled sequences (range=0-30) |
|  | Trail Making test from D-KEFS (Delis et al., 2001) | Shifting | The participant was presented with circles containing letters and numbers. First, the participant is asked to connect the circles containing numbers in numerical order (TMT part 2), after which the participant is asked to alternate between numbers and letters according to numerical and alphabetical order (TMT part 4). | The difference time (seconds) between TMT part 4 and TMT part 2, also defined as shifting cost |
|  | Color-Word interference test from D-KEFS (Delis et al., 2001) | Inhibition | The participant completed three conditions. The first condition required the participant to name out-loud a set of red, blue or green color patches as quickly as possible. In the second condition, the participant read out-loud words presented in black ink, i.e. the words were red, blue and green. In the third condition, the participant is required to state out-loud the printed color of a word, whilst the word itself is an incongruent color (e.g. the word red, printed in a green color). | Time in seconds to complete the third condition minus the second condition, also defined as the inhibition cost |
| Far transfer tests | Selective Reminding Test (Buschke, 1973) | Episodic Memory | This test consisted of an in-house developed list of 18 concrete nouns. The words were presented on a computer screen at the rate of five seconds per item, followed by a free-recall test. On all subsequent trials, words that were not successfully recalled were presented verbally to the participant, followed by another free recall of the entire list. This procedure was repeated four times. | Total number of correct recalled items across all four trials (range=0-72) |
|  | Matrix Reasoning (WAIS-IV) | Problem Solving | The participant is presented with a series of visual stimuli that are arranged in a grid or line and a question mark is placed at the location of the missing stimulus. The participant is requested to choose one of 5 other visual stimuli to complete the above-mentioned visual stimuli. | Total number of correct responses (range=0-26) |
|  | Coding (WAIS-IV) | Speed of Processing | The participant is asked to draw as many symbols as possible into empty boxes, according to a number coding key. | Number of correct responses within 90 seconds (range=0-135) |
|  | Trail Making test from D-KEFS (Delis et al., 2001) | Speed of Processing | The participant is asked to connect the circles containing numbers in numerical order (TMT part 2) | Time to complete TMT part 2 (max=150) |
| Questionnaires | Prospective Retrospective Memory Questionnaire (Crawford et al., 2003) | Subjective cognitive complaints | This questionnaire consists of 16 items on memory slips in everyday life, eight on prospective memory failures (e.g. ‘‘Do you decide to do something in a few minutes time and then forget to do it?’’) and eight on retrospective memory failures (e.g. ‘‘Do you fail to recognize a place you have visited before?’’). Answers were given on a 5-point Likert scale with 1=never and 5=very often. | Total score (range=16-80). |
|  | Hospital Anxiety Depression scale (Zigmond & Snaith, 1983) | Symptoms of depression and anxiety | This questionnaire consists of 7 items on depression and 7 items on anxiety that are scored on a 4-point Likert scale (0-3). | Total score (range=0-21) |
|  | Parkinson’s Disease Questionnaire (Jenkinson et al., 1997) | Function and wellbeing related to PD | This questionnaire consists of 39 items covering the following areas: mobility, activities of daily living, emotional well-being, stigma, social support, cognition, communication and bodily discomfort. The participant indicates the frequency of a certain event by answering on a Likert scale with alternatives never/occasionally/sometimes/often/always or cannot do at all. | Total score and score per subscale |
|  | Checklist Individual Strength (CIS) (Worm-Smeitink et al., 2017) | Fatigue | This questionnaire measures four dimensions of fatigue, i.e. fatigue severity, concentration difficulties, motivation and activity. The participant indicates to which extent they agree with a certain statement through answering on a 7-point Likert scale. | Total score and score per subscale |

**Supplemental material: Description of the Working Memory Updating Training**

The training incorporated six tasks. The first part of the training consisted of the criterion training test, see Figure 1 part 1. In this running span task, named the Letter Memory test (Morris & Jones, 1990), the participant was presented with ten counterbalanced lists of the letters A-D with varied lengths (5-15 letters). The letters were presented one at a time, two seconds per letter. Instructions were to recall the four last presented letters in the correct order when the list presentation ended. The participant responded on a computer keyboard using four adjacent keys with the corresponding letters taped over the keys, i.e. A=index finger, B=middle finger, C=ring finger, D=little finger. This task was not adaptive to the performance of the participant.

The second part of the training consisted of five adaptive running span training tasks similar to the criterion training test, see Figure 1, parts 2a-e. These training tasks will be referred to as ‘running span training task with xxx stimuli’. In four tasks, the participant is presented with five lists of items and asked to recall the last four presented items. The lists were made up of letters (2a), colors (2b), spatial locations (2c) and numbers (2d). The length of these lists varied to be sufficiently demanding throughout the training period with low (4-7 items), medium (6-11 items) and high (5-15 items) cognitive demands. All items were presented sequentially for a duration of 2 seconds per stimulus with an inter-stimulus interval of 1 second. When a participant scored over 80% correct in a certain training task, they advanced to a higher level. The participant responded on a computer keyboard using four adjacent keys with the corresponding stimuli taped over the keys or clicking on the correct stimuli on the computer screen (e.g. for the running span training task with spatial locations as stimuli). Task 2e differs slightly from the other training tasks as participants were instructed to mentally place a list of words into different semantic categories that were presented on the screen by boxes labelled with different categories (i.e. animals, professions, countries, clothes, relatives and sports). After the presentation, the participant was requested to type the last presented word in each category under the matching box. Here, difficulty levels consisted of low (three target categories), medium (four target categories) and high (five target categories). Two versions of this task were used in each training session.

In the case-study, the first 16 training sessions consisted of the following tasks: the criterion training test and the running span training tasks with colors, spatial locations, categories and letters as stimuli (see Figure 1, part 1, 2a-c, 2e). Total training time was 45 minutes. After 16 sessions, an adaptation was made to the training program in order to reduce the total time spent training per day. Therefore, the last 14 sessions consisted of the same training tasks, yet for a total training time of 20 minutes, making the training more enjoyable, motivating and feasible to complete according to FL. Responses were provided through typing or clicking on the correct stimuli on the computer screen. The research team was able to monitor FL’s training progression online.

**Supplemental material: Results from goal-directed movement task**

No significant side effect was found for onset latency *(p =* 0.14), yet a somewhat shorter latency was observed for the right-hand latency (*M* = 286 ms, *SD* = 143 ms) compared to the left hand (*M* = 335 ms, *SD* = 144 ms) independent of test. A significant main effect of side was also observed for both wrist (*M^right^*= 10.3, *SD* = 5.5, *M^left^* = 15.6, *SD*= 7.4), *F*(1, 56) = 12.82, *p* = 0.001, and for index finger (*M^right^* = 15.3, *SD*= 8.1; *M^left^* = 23.1, *SD* = 10.6), *F*(1, 56) = 13.25, *p* = 0.001, displaying a higher number of MUs for the left-sided hand/finger movements (independent of test sessions). In addition, a significant main effect of task was observed for wrist (*M^uni^* = 10.5, *SD* = 7.2; *M^bi^* =16.4, *SD* = 6.0), *F*(1, 56) = 10.53, *p* = 0.002 and for index finger (*M^uni^* = 16.1, *SD* = 10.9; *M^bi^* = 22.3, *SD* = 8.2), *F*(1, 56) = 8.49, *p* = 0.005, showing more MUs during bi-manual tasks compared to uni-manual tasks (independent of test). There were no significant changes observed regarding MUs for elbows, shoulders and head. Nor any significant test session x side interactions were found (*p* > .05), yet graphs visually depict a potential larger improvement at post-test for the more affected side, i.e. the left side.
